# Editorial: Small molecules and smart drug delivery systems for combination cancer immunotherapy

**DOI:** 10.3389/fchem.2024.1412624

**Published:** 2024-04-16

**Authors:** Claudia Ferroni, Greta Varchi

**Affiliations:** Institute for Organic Synthesis and Photoreactivity (ISOF), National Research Council of Italy (CNR), Bologna, Italy

**Keywords:** cancer, immunotherapy, combined treatment, targeted therapy, delivery systems

Cancer immunotherapy has revolutionized cancer treatment by harnessing the power of the immune system to fight tumours. Over the years, significant advancements have been made in understanding tumour biology, immune evasion mechanisms, and the development of novel therapeutic strategies. This editorial highlights recent research contributions focusing on small molecules and smart drug delivery systems for combination cancer immunotherapy.

Ovarian cancer remains a formidable challenge due to high mortality rates and limited treatment options. The study by Liu et al. sheds light on the role of CDK4/6 in ovarian cancer progression, demonstrating that CDK4/6 overexpression correlates with poor patient prognosis. In this scenario, CDK4/6 inhibitors have emerged as promising therapeutic agents in ovarian cancer by promoting antitumour immunity. Moreover, Liu et al. research suggests that the LRRC75A-AS1-hsa-miR-330-5p axis might be responsible for CDK4/6 upregulation, identifying it as a potential therapeutic target, and offering insights into novel treatment strategies for ovarian cancer.

Historically, quantifying the impact of regulatory T cells (Tregs) on tumour growth has been challenging and targeting Tregs presents another avenue for enhancing cancer immunotherapy. By leveraging the selective expression of folate receptor delta (FRδ) on Tregs, Alfar et al. study introduces a novel approach utilizing FRδ-targeted delivery of a potent TLR7 agonist to tumour-resident Tregs. This targeted approach results in a substantial reduction in tumour growth without systemic toxicities, offering a promising strategy for overcoming Treg-mediated immunosuppression in the tumour microenvironment.

In hepatocellular carcinoma (HCC), immunotherapy holds promise as a potential treatment approach. Li et al. bibliometric study provides a comprehensive overview of the evolving landscape of HCC immunotherapy research. With a significant increase in publications in recent years, there is growing interest in exploring immunotherapeutic strategies for HCC treatment. The study highlights emerging trends, prominent research institutions, and future directions for advancing HCC immunotherapy. Notably, combination therapies involving checkpoint inhibitors have shown promise in enhancing anti-tumour immunity and represent a potential future trend for HCC treatment.

Reid et al. report the results of a phase 1 study on the use of RRx-001 + nivolumab in patients with advanced metastatic cancer. By targeting multiple inhibitory pathways, such as PD-1 and CD47, combination therapies aim to overcome resistance mechanisms and improve treatment outcomes. The study underscores the potential of dual checkpoint blockade in traditionally non-responsive tumour types, paving the way for further investigation into combination immunotherapy strategies.

Furthermore, smart drug delivery systems play a crucial role in optimizing the efficacy and safety of cancer immunotherapy. The study by Grindel et al. explores the combination of TLR-9 agonist CpG, emulsified with Lipiodol, with systemic anti-PD-1 for colorectal cancer treatment. By leveraging Lipiodol as a delivery system, the study demonstrates enhanced antitumoral effects and systemic immune responses, highlighting the importance of innovative drug delivery approaches in cancer immunotherapy.

In conclusion, the recent advancements in this field offer ground-breaking opportunities for improving cancer immunotherapy outcomes. By targeting key immune checkpoints, modulating regulatory T cells, and leveraging innovative drug delivery technologies, researchers are paving the way for more effective and personalized cancer treatment approaches. Collaborative efforts between researchers, clinicians, and industry partners are essential for translating these discoveries into clinical practice and ultimately improving patient outcomes in the fight against cancer.

